# Extubation on the Operating Table in Pediatric Cardiac Surgery: A Multicenter Analysis of 986 Patients

**DOI:** 10.1007/s00246-025-03920-7

**Published:** 2025-06-18

**Authors:** Mustafa Kemal Avşar, Yasin Güzel, İbrahim Özgür Önsel, Barış Kırat

**Affiliations:** 1 Division of Pediatric Cardiac Surgery, Department of Cardiovascular Surgery, Faculty of Medicine, Çukurova University, Adana, Turkey; 2 Department of Anesthesiology and Reanimation, Medicana International Istanbul Hospital, Istanbul, Turkey

**Keywords:** Pediatric cardiac surgery, On-table extubation, Reintubation, Congenital heart disease, Early recovery, ICU stay

## Abstract

Extubation on the operating table is increasingly utilized to minimize ventilator-associated complications and promote early recovery in pediatric cardiac surgery. However, its safety across diverse congenital heart disease (CHD) populations remains insufficiently defined. To evaluate the feasibility, safety, and clinical outcomes of on-table extubation across a broad spectrum of corrective and palliative congenital heart surgeries in children. This retrospective multicenter study included 986 pediatric patients (aged 7 days to 16 years) who underwent on-table extubation after CHD surgery between 2019 and 2025. Patients were grouped as corrective (*n* = 632) or palliative (*n* = 354) cases. Primary outcomes were reintubation and mortality. Secondary outcomes included ICU and hospital stay durations, and incidence of ventilator-associated pneumonia (VAP). Overall reintubation and mortality rates were 5.78 and 1.22%, respectively. Corrective procedures demonstrated significantly lower reintubation (4.11%) and mortality (0.63%) compared to palliative surgeries (8.76 and 2.26%, respectively; *p* < 0.01 and *p* < 0.05). Highest complication rates were observed in HLHS (reintubation and mortality 40%) and aortopulmonary shunt (53.13 and 21.88%). In contrast, Glenn and Fontan procedures showed low reintubation (1.69, 2.91%) and minimal mortality. No cases of VAP were reported. Mean ICU and hospital stays were 3.69 and 9.7 days. Of the 57 reintubation events, 23 (40.4%) occurred within 6 h of extubation, suggesting extubation failure, while 34 (59.6%) occurred between 6 and 24 h, potentially due to secondary complications. Early reintubations (0–6 h) were more common in aortopulmonary shunt (17 cases) and coarctation/IAA repair (3 cases), whereas later reintubations (6–24 h) predominated in ToF (5 cases), truncus arteriosus (4 cases), and TGA (3 cases). A moderate correlation was found between reintubation and mortality (Spearman’s *r* = 0.45, *p* < 0.01). On-table extubation is a safe and feasible strategy in pediatric cardiac surgery, particularly in corrective procedures and select single-ventricle palliation. However, caution is warranted in high-risk physiologies such as HLHS and shunt-dependent circulation. Careful perioperative evaluation remains essential for optimal outcomes.

## Introduction

Congenital heart disease (CHD), affecting approximately 0.8–1.2% of live births, remains one of the leading causes of pediatric morbidity and mortality worldwide [1]. Although surgical advancements have markedly improved survival rates, postoperative management continues to significantly impact outcomes in this vulnerable population [2]. Among these, mechanical ventilation plays a pivotal role, but its prolonged use is associated with well-documented risks, including ventilator-associated pneumonia (VAP), increased length of stay, sedation-related complications, and elevated healthcare costs [3, 4]. To address these challenges, early extubation strategies have been increasingly advocated. In particular, intraoperative extubation—defined as removal of the endotracheal tube in the operating room immediately after skin closure—has emerged as a potentially safe and efficient practice in selected patients. The return to spontaneous respiration enhances venous return, improves cardiac output, and lowers pulmonary vascular resistance. However, in conditions such as low cardiac output syndrome (LCOS) or heart failure, positive pressure ventilation may support cardiac output by reducing systemic ventricular workload [5]. Several studies have demonstrated that, in low-risk corrective surgeries, intraoperative extubation may reduce ICU stay by up to 18 h and decrease overall hospital length of stay by 20–30% [6–9].

Beyond its role in mitigating ventilator-associated complications, intraoperative extubation offers a multidimensional array of physiological and clinical advantages that align with modern principles of enhanced recovery in pediatric cardiac surgery [6, 8]. Respiratorily, the early restoration of spontaneous breathing facilitates more physiological ventilation mechanics, thereby improving alveolar recruitment, enhancing mucociliary clearance, and reducing the risk of atelectasis and ventilator-induced lung injury [3, 10]. From a hemodynamic perspective, spontaneous ventilation reduces intrathoracic pressure, augments venous return, and optimizes cardiac output—effects particularly beneficial in patients with passive pulmonary circulation, such as those undergoing Glenn or Fontan procedures [11, 12]. Furthermore, early extubation expedites the initiation of enteral feeding, thereby preserving gastrointestinal mucosal integrity and promoting mesenteric perfusion, which are known to decrease the incidence of postoperative ileus and feeding intolerance [13, 14]. The capacity for earlier mobilization following extubation contributes to the prevention of ICU-acquired weakness and enhances functional recovery trajectories [14, 15]. On a psychosocial level, prompt awakening and restoration of spontaneous interaction enhance neurobehavioral stability and facilitate early parental bonding, both of which have been independently associated with improved family centered outcomes in pediatric intensive care [16, 17].

Nevertheless, despite these multifaceted benefits, the safety and feasibility of intraoperative extubation across the full spectrum of CHD patients remain subjects of debate. Patients undergoing complex repairs or palliative procedures frequently possess limited cardiopulmonary reserve and are prone to complications such as pulmonary hypertension and low cardiac output syndrome, both of which may increase the risk of extubation failure. Indeed, reintubation rates of up to 10.2% have been reported in palliative cohorts [11, 16].

Conversely, favorable outcomes have been observed in selected single-ventricle patients, particularly those undergoing Glenn or Fontan procedures, where intraoperative extubation has been associated with reductions in pleural effusions, inotrope use, and ICU stay [11, 12, 18]. These conflicting findings highlight the need for large-scale, comparative data to better define patient selection criteria and refine perioperative strategies.

In this multicenter retrospective study, we evaluated the safety and efficacy of intraoperative extubation in 986 pediatric patients undergoing CHD surgery between April 2019 and January 2025. By comparing outcomes between corrective (*n* = 632) and palliative (*n* = 354) procedures, we aim to contribute to evidence-based perioperative planning and enhance protocol-driven extubation practices in pediatric cardiac surgery.

## Materials and Methods

### Study Design and Patient Population

This multicenter, retrospective cohort study included 986 pediatric patients who underwent cardiac surgery for congenital heart disease (CHD) between April 2019 and January 2025. The procedures were performed at four tertiary care centers in Turkey: Çukurova University Faculty of Medicine Balcalı Hospital (Adana), Medicana International Hospital (Istanbul), Emsey Hospital (Istanbul), and Atlas University Medical Hospital (Istanbul).

Patients ranged in age from 7 days to 16 years (mean: 3.9 years) and in weight from 2.9 to 43 kg (mean: 5.4 kg). Based on surgical intent, patients were categorized into two groups: corrective procedures (*n* = 632) and palliative procedures (*n* = 354) (Table 1). All patients who were successfully weaned from cardiopulmonary bypass (CPB), regardless of inotropic support requirement, and those who underwent surgery without CPB were considered eligible for intraoperative extubation.

**Table 1:** Patient distribution and outcomes in corrective and palliative surgical groups

Diagnosis	Total Patients	Corrective (*n*)	Palliative (*n*)	Reintubation (%)	Mortality (%)
VSD closure ± aortic arch	121	121	0	1.65	0
AVSD complete repair	94	94	0	6.38	1.03
ToF complete repair	107	107	0	4.67	0
TGA (Jatene)	32	32	0	9.38	3.13
TAPVD complete repair	49	49	0	12.24	4.08
ASD closure	17	17	0	0	0
Coarctation or interrupted aortic arch	28	28	0	10.71	0
Truncus arteriosus complete repair	16	16	0	25.00	0
Conduit replacement	58	58	0	0	0
Aortic/mitral valve repair/replacement	65	65	0	1.54	0
Subaortic stenosis (Morrow/Konno)	45	45	0	2.22	0
Ross procedure	11	11	0	0	0
ALCAPA or coronary anomaly repair	8	8	0	0	0
Aortopulmonary shunt ± unifocalization	32	0	32	53.13	21.88
Pulmonary artery banding	52	0	52	5.77	1.92
Glenn procedure	118	0	118	1.69	0
Fontan surgery	103	0	103	2.91	0.97
Brock or Sano shunt	38	0	38	15.79	5.26
Palliative switch	6	0	6	16.67	16.67
HLHS (Norwood or bilateral PA banding)	5	0	5	40.00	40.00
Total	986	632	354	5.78	1.22

Exclusion criteria included preoperative mechanical ventilation, persistent metabolic acidosis (arterial pH < 7.25), or intraoperative hemodynamic instability that precluded safe extubation attempts.

### Anesthetic and Surgical Protocol

A standardized anesthesia and extubation protocol was employed across all centers, including short-acting agents and structured weaning criteria. The protocol details, including induction agents, intraoperative monitoring, analgesia, and extubation sequence, are summarized in Table 2. General anesthesia was induced using short-acting agents: remifentanil (0.1–0.5 µg/kg/min infusion), propofol (2–3 mg/kg bolus at induction; 1–2 mg/kg as maintenance boluses), and rocuronium (0.6–1 mg/kg bolus) for neuromuscular blockade. Neuromuscular reversal was achieved with sugammadex (2–3 mg/kg IV) prior to extubation.

**Table 2:** Summary of anesthesia and extubation protocol

Stage	Procedure	Dosage	Notes
Preoperative	IV access, Midazolam	0.03 mg/kg	For amnesia during family separation
Monitoring	ECG, pulse oximetry, non-invasive BP	–	Initiated in operating room
Induction	Propofol, Rocuronium	2–3 mg/kg, 1 mg/kg	For orotracheal intubation
Maintenance	Sevoflurane	MAC 0.5–2	2–3% during CPB
Neuromuscular blockade	Rocuronium	0.2 mg/kg	Paused during hypothermia unless necessary
Additional anesthesia	Propofol	1–2 mg/kg	For sternotomy and post-bypass depth
Controlled hypotension	Nitroglycerin	IV, titrated	Upon surgeon request
Analgesia	Pethidine, Paracetamol	0.4, 15 mg/kg	At sternal cerclage
Extubation preparation	Sevoflurane cessation, O_2_ flow	15 L/min	5–10 min before skin closure
Extubation	Sugammadex	2–3 mg/kg	Removed upon full awakening
Postop monitoring	Venturi mask oxygen, Hemodynamic monitor	–	2–5 min before ICU transfer
ICU analgesia	Pethidine	0.3 mg/kg	Additional dose as needed
Extubation exclusion	–	–	If high inotrope need or severe acidosis

Standard surgical techniques for CHD repair or palliation were employed according to institutional protocols. Intraoperative monitoring included electrocardiography, arterial blood pressure, central venous pressure, pulse oximetry, capnography, and near-infrared spectroscopy (NIRS) when indicated (Table 3).

**Table 3:** ICU and hospital stay durations

Parameter	Mean duration (days)
ICU stay	3.69
Hospital stay	9.7

### Extubation Criteria and Postoperative Transfer

Extubation was performed on the operating table, immediately after skin closure, provided the following criteria were met:

1. Hemodynamic stability (mean arterial pressure > 50 mmHg and age-appropriate heart rate)
2. Effective spontaneous respiration (respiratory rate > 12/min, SpO_2_ > 90% on FiO_2_ < 0.5)
3. Normocapnia and adequate acid–base status (arterial pH > 7.35, PaCO_2_ < 45 mmHg)

After extubation, patients were observed for 5–10 min in the operating room for respiratory and hemodynamic adequacy before being transferred to the intensive care unit.

### Data Collection and Statistical Analysis

Demographic, surgical, and outcome data were retrieved from electronic medical records of the participating centers. Primary outcome measures included reintubation and mortality. Secondary outcomes were ventilator-associated pneumonia (VAP), length of ICU stay, and total hospital stay.

VAP was diagnosed according to the CDC/NHSN surveillance definition for pediatric ventilator-associated events, updated in 2020 [19]. Continuous variables were expressed as mean ± standard deviation and compared using the Student’s *t*-test. Categorical variables were analyzed using the chi-square test. The correlation between reintubation and mortality was evaluated using Spearman’s rank correlation. A *p*-value < 0.05 was considered statistically significant.

All analyses were performed using IBM SPSS Statistics version 26. No formal sample size calculation was performed due to the retrospective design. The study was approved by the Çukurova University Ethics Committee, and the requirement for informed consent was waived.

## Results

A total of 986 pediatric patients underwent intraoperative extubation following congenital heart disease (CHD) surgery between April 2019 and January 2025. Of these, 632 patients (64.1%) underwent corrective procedures and 354 patients (35.9%) underwent palliative procedures. The overall reintubation rate was 5.78% (*n* = 57), and the overall in-hospital mortality rate was 1.22% (*n* = 12).

### Corrective Surgery Group

Among the 632 patients in the corrective group, reintubation was required in 26 cases (4.11%), and mortality occurred in 4 patients (0.63%). The highest reintubation rates were observed in patients undergoing:

1. Coarctation/interrupted aortic arch repair (17.86%; 5/28)
2. TGA (Jatene procedure) (12.5%; 4/32)
3. TAPVD repair (6.12%; 3/49)

**Fig. 1:**
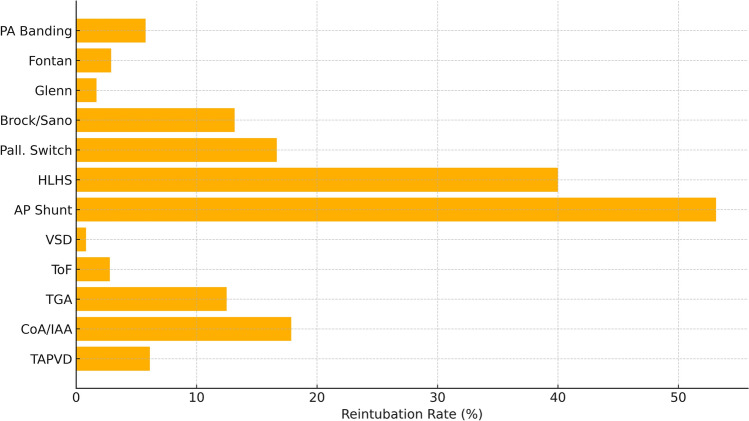
Reintubation rates by diagnosis. Bar chart showing the percentage of reintubation across major diagnostic categories in the study population. Aortopulmonary shunt and HLHS patients had the highest reintubation rates among all subgroups

Notably, TOF repair (*n* = 107) had a low reintubation rate of 2.8% (3 patients) with no mortality, and VSD closure ± arch repair had only one reintubation event (0.83%) and no deaths. No reintubation or mortality was observed following ASD closure, conduit replacement, Ross procedure, coronary anomaly repair, or valve surgery. A full breakdown of diagnosis-specific reintubation rates is presented in Fig. 1.

**Fig. 2:**
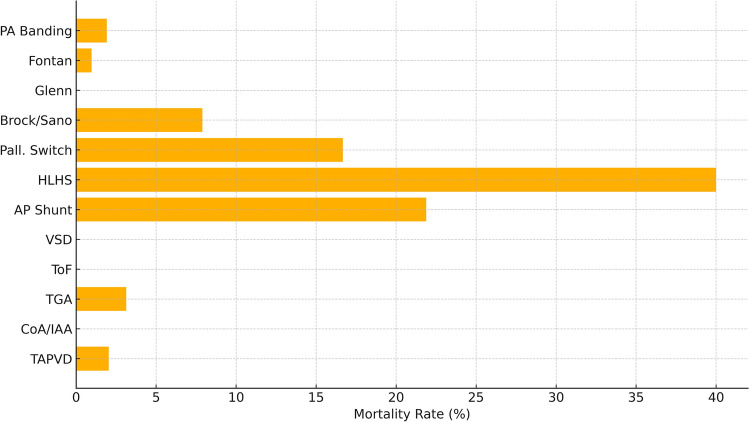
Mortality rates by diagnosis. Distribution of in-hospital mortality rates by diagnostic category. Highest mortality was observed in patients with HLHS and aortopulmonary shunts

Mortality within the corrective group was confined to patients with TGA (1 death), TAPVD (1 death), and AVSD (1 death). The distribution of mortality rates by diagnosis is illustrated in Fig. 2. The primary causes of mortality in the corrective group were low cardiac output syndrome (LCOS) in the TGA patient, reactive pulmonary hypertension in the TAPVD patient, and LCOS and postoperative malignant arrhythmia in the two AVSD patients, respectively.

### Palliative Surgery Group

Among the 354 patients undergoing palliative surgery, reintubation was observed in 31 patients (8.76%) and mortality in 8 (2.26%), both significantly higher than in the corrective group (*p* < 0.01 and *p* < 0.05, respectively). In the palliative group, mortality in aortopulmonary shunt cases was primarily due to overflow (four cases), pulmonary edema (two cases), and sudden cardiac arrest (one case). For other palliative procedures, mortality was attributed to low cardiac output syndrome (LCOS) in two HLHS cases, pulmonary hypertensive crisis in one palliative switch case, and arrhythmia in one Brock/Sano shunt case.

The highest reintubation and mortality rates were observed in:

1. Aortopulmonary shunt ± unifocalization (reintubation 53.13%, mortality 21.88%)
2. HLHS (Norwood or bilateral PA banding) (reintubation and mortality 40%)
3. Palliative arterial switch (reintubation and mortality 16.67%)
4. Brock/Sano shunt (reintubation 13.16%, mortality 7.89%)

In contrast, favorable outcomes were recorded in:

1. Glenn procedure (reintubation 1.69%, no mortality)
2. Fontan procedure (reintubation 2.91%, mortality 0.97%)
3. Pulmonary artery banding (reintubation 5.77%, mortality 1.92%)

**Fig. 3:**
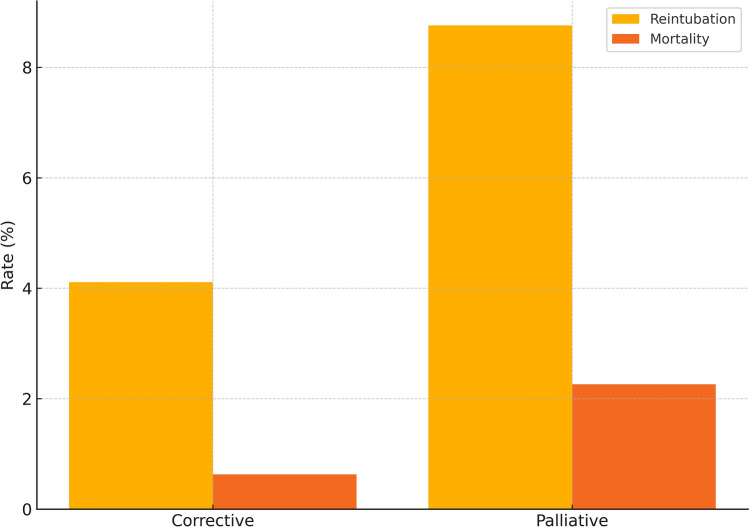
Groupwise comparison of reintubation and mortality. Comparative bar chart of reintubation and mortality rates between corrective and palliative surgical groups. Statistically significant differences were observed, favoring corrective procedures (*p* < 0.01 for reintubation; *p* < 0.05 for mortality)

Groupwise comparison of reintubation and mortality rates between corrective and palliative cohorts is presented in Fig. 3.

Demographic and clinical characteristics are summarized in (Table 4). Neonates (< 30 days) exhibited significantly higher reintubation rates, particularly in corrective procedures (e.g., coarctation/IAA, TGA, TAPVR) and palliative procedures (e.g., HLHS, aortopulmonary shunt), compared to older children (*p* < 0.01).

**Table 4:** Patient characteristics by diagnosis, surgical type, and reintubation status

Diagnosis	Surgical type	Reintubated (*n*)	Median age (days)	Median weight (kg)	CPB time (min)	Cause of reintubation	Time to reintubation (h)
VSD + Arch repair	Corrective	2	45	4.8	82	Atelectasis/Laryngeal edema	0–6
ToF repair	Corrective	5	180	6.3	90	Atelectasis/Phrenic nerve injury/Reoperation	6–24
TGA (Jatene procedure)	Corrective	3	7	3.1	98	Low cardiac output/Myocardial dysfunction	6–24
TAPVD repair	Corrective	3	14	3.5	78	Pulmonary edema/Phrenic nerve injury	6–24
Coarctation/IAA repair	Corrective	3	12	3.2	65	Laryngeal edema	0–6
Truncus Arteriosus Repair	Corrective	4	20	3.3	105	Prolonged sedation/Pulmonary edema	6–24
Aortic/mitral valve repair/repl	Corrective	1	300	7.5	63	Postoperative bleeding/Prolonged sedation	6–24
Subaortic stenosis (Morrow/Konno)	Corrective	1	240	6.5	57	Residual gradient/Low cardiac output	6–24
Aortopulmonary Shunt ± Unifoc	Palliative	17	20	3.0	60	Laryngeal edema/Pulmonary edema/Sudden cardiac arrest	0–6
Pulmonary artery banding	Palliative	3	30	3.5	40	Hypercarbia/Respiratory acidosis	6–24
Glenn procedure	Palliative	2	180	6.0	75	Pleural effusion/Diaphragmatic dysfunction	0–6
Fontan surgery	Palliative	3	900	21.0	80	Laryngeal edema	6–24
Brock or Sano Shunt	Palliative	6	10	2.8	55	Desaturation/Diaphragm paresis	6–24
Palliative arterial switch	Palliative	1	14	3.2	90	Laryngeal edema	6–24
HLHS (Norwood or bilateral PA)	Palliative	2	5	3.0	138	Residual pulmonary hypertension	6–24

### ICU and Hospital Stay

**Fig. 4:**
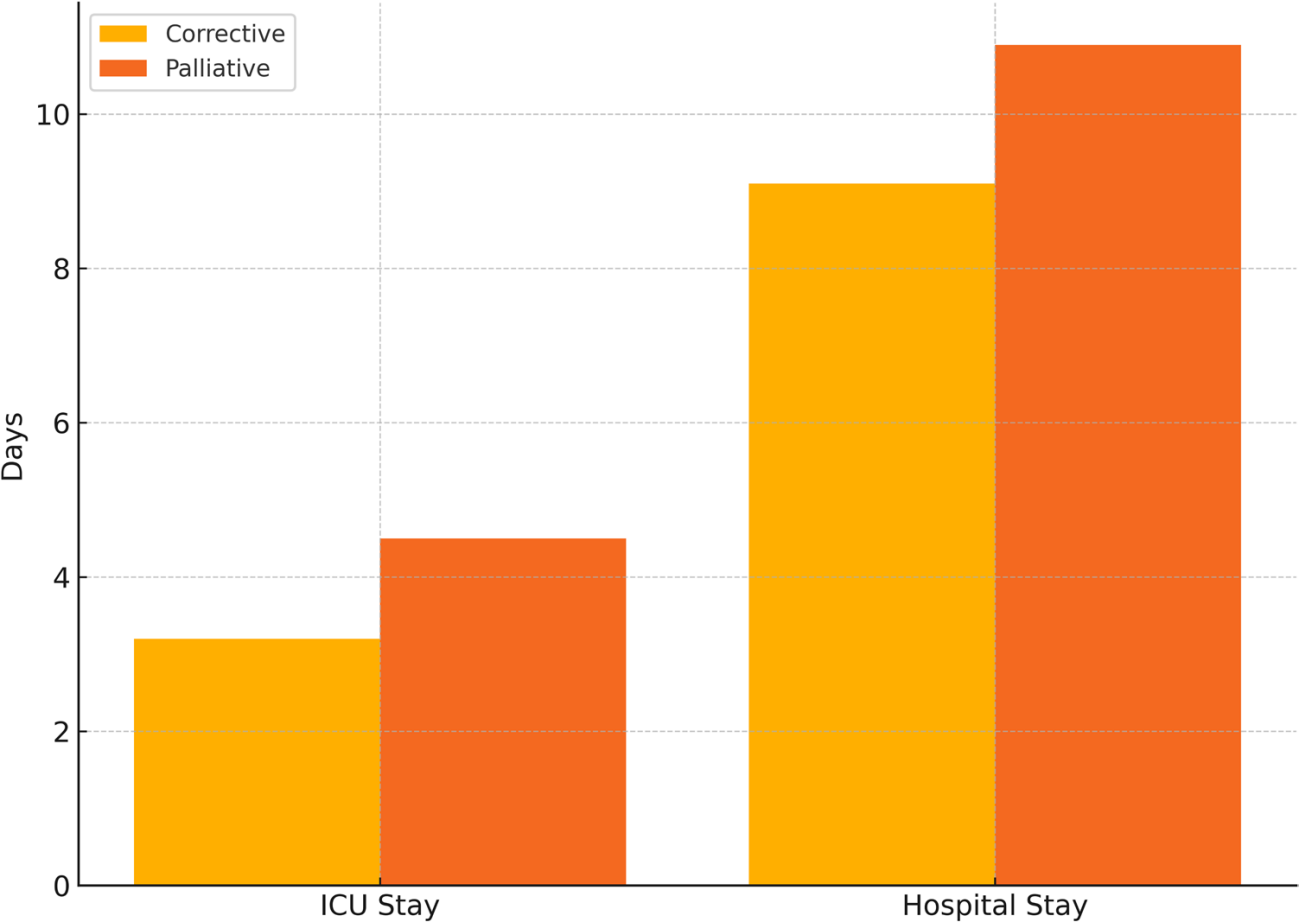
ICU and hospital stay by surgical group. Mean ICU and total hospital stay durations in days for corrective and palliative surgery groups. Palliative patients had longer ICU and hospital stays (*p* < 0.01)

The overall mean ICU stay was 3.69 ± 1.5 days, and the mean hospital stay was 9.7 ± 2.8 days. When stratified by surgical group, palliative patients had significantly longer ICU (4.5 vs. 3.2 days) and hospital stays (10.9 vs. 9.1 days) compared to corrective patients (*p* < 0.01) (Fig. 4).

No cases of ventilator-associated pneumonia (VAP) were observed in the entire cohort.

### Correlation Between Reintubation and Mortality

**Fig. 5:**
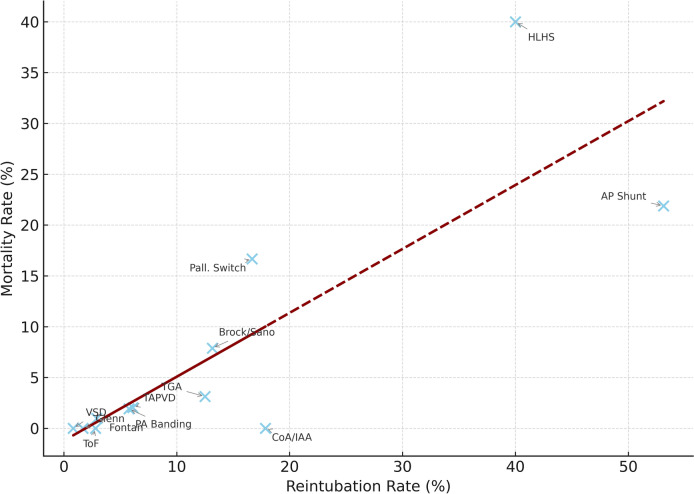
Correlation between reintubation and mortality rates. Scatter plot demonstrating a positive correlation (Spearman’s *r* = 0.45, *p* < 0.01) between reintubation and mortality rates by diagnosis. Regression line is shown for reference

A moderate positive correlation was found between reintubation and mortality rates across diagnostic subgroups (Spearman’s *r* = 0.45, *p* < 0.01). The relationship was most pronounced among high-risk palliative procedures such as HLHS and aortopulmonary shunt patients. This correlation is visually summarized in Fig. 5.

## Discussion

This multicenter retrospective study demonstrates that extubation on the operating table is both feasible and safe in a broad pediatric population undergoing congenital heart surgery. With an overall reintubation rate of 5.78% and mortality of 1.22%, our results are consistent with previously published early extubation experiences in pediatric cardiac surgery [5, 8]. Among corrective procedures, coarctation/IAA repair (17.86%), TGA repair (12.5%), and TAPVR repair (6.12%) exhibited elevated reintubation rates, likely attributable to the neonatal age of these patients (median age 7–14 days) and procedure-specific complications. Neonates, with limited cardiopulmonary reserve, are particularly susceptible to complications such as laryngeal edema, pulmonary edema, or low cardiac output. For instance, TGA repair may result in myocardial dysfunction following the arterial switch procedure, increasing the risk of respiratory failure [4]. Similarly, TAPVR repair often involves pulmonary vein anomalies, leading to pulmonary edema or phrenic nerve injury, which contribute to extubation failure [10]. Coarctation/IAA repair frequently presented with laryngeal edema, potentially linked to the delicate airways of neonates [5, 20]. These findings underscore the need for cautious evaluation of on-table extubation in neonates. While spontaneous ventilation typically enhances venous return and cardiac output, positive pressure ventilation may stabilize hemodynamics in patients with low cardiac output syndrome or severe heart failure by reducing ventricular preload and afterload [5].

Our subgroup analysis revealed a significant difference between corrective and palliative surgeries. In the corrective cohort, reintubation (4.11%) and mortality (0.63%) rates were low, supporting the safety of on-table extubation in hemodynamically stable patients following definitive repair. These findings align with prior studies demonstrating the physiologic advantages of early extubation—enhanced venous return, improved cardiac output, and shorter ICU stays—especially in lower-risk patients [21, 22].

In contrast, the palliative group demonstrated higher reintubation (8.76%) and mortality (2.26%) rates, with specific diagnoses such as HLHS (40% reintubation and mortality) and aortopulmonary shunt (53.13% reintubation, 21.88% mortality) exhibiting the highest complication profiles. The primary causes of mortality in our cohort reflect the physiological challenges of specific procedures. In the corrective group, deaths were primarily due to low cardiac output syndrome (LCOS) in TGA and AVSD cases, reactive pulmonary hypertension in TAPVD, and malignant arrhythmia in one AVSD case, highlighting the complexity of neonatal repairs [4]. In the palliative group, aortopulmonary shunt mortality was driven by overflow (four cases), pulmonary edema (two cases), and sudden cardiac arrest (one case), while LCOS, pulmonary hypertensive crisis, and arrhythmia predominated in HLHS, palliative switch, and Brock/Sano shunt cases, respectively, underscoring the fragility of shunt-dependent circulation and the risk of hemodynamic instability [10]. These findings emphasize the need for tailored postoperative management to mitigate these risks in high-risk populations. These outcomes underscore the limitations of on-table extubation in patients with severely compromised cardiopulmonary physiology and shunt-dependent circulation.

Several prior studies have raised similar concerns. Gupta et al. reported that systemic-to-pulmonary artery shunt patients were particularly prone to extubation failure due to unstable pulmonary blood flow and risks of shunt overcirculation, especially in the presence of low diastolic pressure or hypotension [4]. Another study emphasized the hemodynamic fragility of shunt-dependent physiology in the early postoperative period, where minimal perturbations in resistance could lead to decompensation [5, 6]. Similarly, Mastropietro et al. cautioned against immediate extubation in neonates with passive pulmonary circulation, noting that positive pressure ventilation may help stabilize early pulmonary blood flow before vascular tone adaptation [11].

Based on our own clinical observations and outcome data, we have modified our institutional protocols. All patients undergoing aortopulmonary shunt or comparable palliative procedures are now electively kept intubated for a minimum of 24 h postoperatively. This delayed extubation strategy allows for hemodynamic assessment, inotrope titration, and real-time evaluation of shunt flow stability. Since the adoption of this protocol, extubation failure in this subgroup has declined.

Conversely, certain palliative procedures particularly Glenn and Fontan operations showed excellent tolerance for on-table extubation, with reintubation rates below 3% and minimal mortality. These findings support earlier reports indicating that the unique physiology of superior cavopulmonary and total cavopulmonary connections may benefit from spontaneous ventilation, which enhances systemic venous return and reduces pleural effusions [11, 12, 16].

An additional noteworthy finding in our study is the absence of ventilator-associated pneumonia (VAP), which we attribute to minimized mechanical ventilation time and optimized extubation timing. This observation reinforces the relevance of early extubation strategies as part of enhanced recovery protocols in pediatric cardiac surgery [13, 23].

In addition to physiologic and respiratory advantages, early extubation appears to offer meaningful benefits in terms of health resource utilization and neurodevelopmental protection. Prior studies have reported significant reductions in ICU length of stay and total hospital costs associated with successful early extubation [13, 14]. These findings are echoed in our own institutional experience, where streamlined recovery following on-table extubation has facilitated more efficient ICU turnover and reduced the need for prolonged sedation or invasive monitoring.

Moreover, minimizing sedation and ventilator exposure in neonates and infants may mitigate the risk of adverse neurodevelopmental outcomes, particularly in patients with syndromic or borderline neurologic profiles [18]. In our practice, earlier awakening after extubation has also enabled timely initiation of physical therapy and environmental stimulation—factors we believe contribute to more favorable long-term recovery.

Nevertheless, on-table extubation may not be appropriate for all patients. In particular, those with limited hemodynamic reserve, pulmonary hypertension, or shunt-dependent physiology may experience circulatory instability following the abrupt loss of positive pressure ventilation. This can disrupt the balance between systemic and pulmonary vascular resistance, potentially compromising cardiac output [11]. Additionally, several studies have shown that failed extubation and subsequent reintubation are associated with increased morbidity and mortality [4].

In our cohort, a moderate positive correlation (*r* = 0.45) was observed between reintubation and mortality, highlighting the need to base extubation decisions not solely on surgical category, but on a patient’s physiological reserve and intraoperative stability. The timing of reintubation provides further insight into its etiology and relationship with mortality. Early reintubations (within 6 h) are more likely to reflect extubation failure due to inadequate respiratory or hemodynamic reserve, as observed in aortopulmonary shunt and coarctation/IAA repair cases. In contrast, later reintubations (6–24 h) may result from secondary complications such as pulmonary edema, myocardial dysfunction, or pleural effusions, as seen in ToF, TGA, and truncus arteriosus cases [4]. This suggests that the association between reintubation and mortality is not solely attributable to early intraoperative extubation but may reflect broader systemic instability, necessitating careful patient selection and postoperative monitoring [10]. Accordingly, in high-risk patients, we now favor a more conservative strategy involving elective postoperative mechanical ventilation for at least 24 h.

On a pharmacologic level, patients extubated on the operating table consistently required lower cumulative doses of opioids and benzodiazepines. While this finding was not formally quantified in the present study, we observed a lower incidence of post-extubation withdrawal symptoms and ICU delirium among these patients, especially in toddlers and young children.

Finally, from an infection control standpoint, the complete absence of ventilator-associated pneumonia (VAP) in our cohort strongly supports the hypothesis that early extubation reduces the opportunity for pulmonary nosocomial infections [19]. While this may reflect careful perioperative protocols, we believe that limiting ventilator duration remains a key contributor.

These cumulative observations suggest that the benefits of on-table extubation extend beyond physiologic recovery to include logistical, developmental, and economic dimensions. However, we acknowledge that these interpretations require prospective validation and standardized outcome tracking in future studies.

Beyond physiological and clinical outcomes, early extubation may offer additional psychosocial advantages by enabling spontaneous awakening and earlier interaction in the immediate postoperative period. These elements are central to modern family centered care models in pediatric intensive care, and have been associated with reduced parental anxiety, enhanced neurodevelopmental stability, and improved overall recovery experience [17].

In our clinical experience, early extubation facilitates improved parental engagement, emotional reassurance, and more positive caregiver-child dynamics. These non-physiologic benefits, while harder to quantify, appear to play a meaningful role in postoperative recovery and should not be overlooked when considering extubation strategies in vulnerable pediatric populations.

Finally, the moderate but significant correlation between reintubation and mortality (Spearman’s *r* = 0.45, *p* < 0.01) highlights the importance of respiratory stability as a key determinant of postoperative outcomes. Reintubation, especially in fragile physiologies, should not be regarded as a benign event but as a signal of systemic imbalance and potential decompensation.

## Limitations

This study has several limitations. First, due to its retrospective design, potential selection bias and inter-institutional variability in patient inclusion and data collection cannot be excluded. Although extubation decisions were based on defined physiological criteria, in practice, they were also influenced by the clinical judgment of the attending anesthesia and surgical teams. This may have introduced subjective variation in extubation practices across procedures.

Additionally, specific physiologic parameters such as sedation depth, respiratory mechanics, and postoperative inotrope scores—which could affect extubation success—were not prospectively standardized or uniformly recorded. Furthermore, detailed etiological analysis of reintubation events (e.g., airway obstruction, pleural effusion, low cardiac output) was not performed; outcomes were instead reported at the diagnostic level. Moreover, the retrospective design limited the granularity of reintubation timing data, with only broad time intervals (0–6 h and 6–24 h) available, potentially restricting a comprehensive analysis of extubation failure versus secondary complications. This limits the ability to fully explore the underlying mechanisms of extubation failure. Despite these limitations, the study has notable strengths. Its multicenter design, large sample size (*n* = 986), and inclusion of both corrective and palliative surgeries enhance the clinical generalizability of the findings. Furthermore, detailed etiological analysis of reintubation events (e.g., airway obstruction, pleural effusion, low cardiac output) was not performed; outcomes were instead reported at the diagnostic level. Additionally, due to the retrospective design, detailed causes of mortality were not uniformly documented for all cases, particularly in the palliative group, where some causes were inferred based on clinical likelihood and literature.

## Conclusion

This large multicenter study demonstrates that extubation on the operating table is a safe and effective strategy in pediatric congenital heart surgery when applied to appropriately selected patients. Corrective procedures and certain stable palliative interventions—such as Glenn and Fontan operations—exhibited high tolerance for this approach and may benefit from enhanced postoperative recovery.

However, in high-risk populations characterized by shunt-dependent physiology or limited cardiopulmonary reserve—particularly in HLHS and aortopulmonary shunt patients—on-table extubation was associated with significantly higher rates of reintubation and mortality. These findings highlight the need for extubation decisions to be guided not only by the type of surgery, but more importantly by the patient’s physiological reserve and intraoperative stability.

Accordingly, in high-risk patients, we now recommend a more conservative approach: elective postoperative mechanical ventilation for a minimum of 24 h to allow for hemodynamic stabilization, shunt flow assessment, and careful titration of inotropic and ventilatory support. In contrast, in physiologically suitable patients, early extubation may reduce ventilator-associated risks, shorten ICU stay, and accelerate recovery.

Extubation on the operating table should not be viewed as a routine measure, but rather as a personalized, physiology-driven strategy. When applied judiciously and selectively, this approach can meaningfully improve perioperative outcomes and postoperative comfort in pediatric cardiac surgery.

## Data Availability

The datasets generated and analyzed during the current study are not publicly available due to institutional privacy regulations but are available from the corresponding author on reasonable request.
